# Scents from the past: Lineage history and terminal identity in the olfactory system

**DOI:** 10.1002/ntls.20220037

**Published:** 2022-08-07

**Authors:** Sriivatsan G. Rajan, Ankur Saxena

**Affiliations:** 1 Department of Biological Sciences, University of Illinois Chicago, Chicago, Illinois, USA; 2 University of Illinois Cancer Center, Chicago, Illinois, USA

**Keywords:** lineage-tracing, neural crest, neurons, olfactory, placode, stem cells

The development of the vertebrate nose depends upon the interaction and intermingling of multiple progenitor subtypes, giving rise to olfactory epithelia that detect sensory information and transmit it to the brain.^1,2^ The olfactory epithelium is a dense and complex structure that houses numerous specialized cell types, including olfactory sensory neurons (OSNs) and, additionally, is thought to be the origin of migratory gonadotropin-releasing hormone (GnRH) neurons.^2–4^

In the context of rapid craniofacial morphogenesis that alters the multicellular landscape, it has proved challenging to label, image, and/or track the behavior of densely packed olfactory cells to identify the sources of OSNs and GnRH neurons. The issue is further compounded by the highly migratory, diverse behaviors of neural crest stem cells (NCCs) as they move toward the anterior end of the embryo.^5,6^ Several approaches across multiple species and using multiple *in vivo* and *in situ* methods have pointed to placodal and/or neural crest (NC) origins for OSNs and GnRH neurons,^2–4,7–16^ and a “placode versus neural crest” debate remains ongoing. Additionally, cellular origins could differ across species, underscoring the need to broaden the model systems and techniques used to probe these discrepancies. Given these factors, as well as variability in how terms such as “preplacodal” and “neural crest precursor” are applied, there remain outstanding questions regarding not just the canonical origins of OSNs and GnRH neurons but also (1) how, where, and when their spatiotemporal origins ought to be defined in a rapidly developing embryo; (2) how much value to place on conservation across model systems; (3) how to bridge the gap between traditional fate-mapping of cellular origins and the actual behavior of progenitor cells and/or their derivatives.

In their paper in *Natural Sciences*, Koontz et al. use retroviral lineage tracing to label olfactory placodal cells and NCCs in chicken embryos and track their contributions to the olfactory system.^17^ They combine lineage analysis with established markers for olfactory ensheathing cells (i.e., olfactory glia), basal stem cells, OSNs, and GnRH neurons, providing a much-needed, comprehensive overview of the origins of key cell types in the chicken olfactory system. Consistent with previous studies in mice and chicken,^4,10,18,19^ their work finds a solely NC origin for olfactory ensheathing cells and a dual placodal/NC origin for basal stem cells. In addition, they discover that basal stem cells derived from both placodal and NC cells express p63, which labels horizontal basal cells, one of the two types of olfactory basal stem cells.^20,21^ It has been previously suggested in mice that, as the olfactory epithelium ages, the proportion of placode-derived horizontal basal cells gradually decreases, while the proportion of NC-derived horizontal basal cells increases.^10^ Thus, it will be useful to investigate whether a similar shift occurs during chicken olfactory development. As for GnRH neurons, conflicting reports across model systems have argued for their derivation from the placode and/or NC.^4,11,13,22^ Here, Koontz et al. show that GnRH neurons can be derived from both cell types, in line with previous findings in mice.^4^ Given that GnRH neurons exhibit significant functional heterogeneity and diversity in expression profiles across species,^23,24^ it will be interesting to see whether future cross-species lineage-mapping of GnRH neuronal subtypes reveals patterns of specification that correlate with distinct functional identities.

Sniffing out the origins of OSNs has also proved challenging. Studies in zebrafish, mouse, and frog olfactory epithelia have yielded contradictory results, with some experiments concluding that OSNs are derived from both the olfactory placode and NC,^3,4,10,15,16^ while others argue that OSNs come from placodal cells only.^8,9,13^ OSN lineages had not been extensively traced in the chicken model system, and thus Koontz et al. offer important insights into a new species, demonstrating that chicken OSNs are derived from both the placode and NC.^17^ Of note, NC-derived OSNs are described here in 7-day old embryos, that is, 5 days after NCCs first arrive to form the mesenchymal nasal cavity,^25^ and are shown to be a small subset of the total population of OSNs. Consequently, it is worth considering whether the NC contribution to OSNs might begin and/or accelerate at time points later than that of the initial placodal contribution. If so, that staggered timing would be consistent with our previous work in zebrafish embryos,^3^ which demonstrated that NCCs start ingressing into the developing olfactory epithelium several hours after the olfactory placode begins generating OSNs. A delay between placodal induction of neurogenesis and subsequent NC contributions could help explain why the latter has been harder to pin down across species.

Koontz et al.’s findings are also supported by intriguing molecular data in the chicken embryo, where Notch signaling inhibition in cranial NCCs has been previously shown to promote their differentiation into ectopic neurons rather than olfactory ensheathing cells.^26^ Therefore, it seems that olfactory system-associated NCCs harbor neurogenic potential that can be activated via the inhibition of a single signaling pathway, and it will be worthwhile to investigate, in multiple species, if NC-derived OSNs can be stimulated or inhibited via modulation of Notch signaling or other pathways.

Almost all lineage tracing studies focused on OSNs and GnRH neurons have chosen to analyze only placodal cells or only NCCs,^3,9,10,13^ leaving open the question of what each individual approach/technique would have found by labeling the other cell type. Koontz et al., on the other hand, simultaneously track both placodal and NC cells, similar to work done previously in mice,^4^ and the addition of chicken to the zebrafish/frog/mouse portfolio of lineage tracing significantly strengthens the broader argument for a pan-species NC contribution to OSNs and/or GnRH neurons. It is important to note that this point of view is not necessarily at odds with evidence for placodal contributions. Previous work has suggested that the neural plate border may contain a more heterogeneous mixture of cells than previously appreciated,^27^ and our earlier findings concluded that a subset of, but not all, microvillous OSNs are NC-derived.^3^ Thus, a dual placode/NC contribution to OSNs now has supporting evidence in four species and is consistent with data from placodal lineage tracing that did not query the possibility of NC contributions.

This story may have more twists and turns ahead. The chicken olfactory system is thought to be devoid of microvillous OSNs, leaving open the question of what type(s) of NC-derived neurons Koontz et al. labeled and whether they have a different functional role than those that are placode-derived. Additionally, a previously undescribed population of rod cells was recently discovered in the zebrafish olfactory pit.^28^ These cells of unknown origin are located near OSNs, do not have axons, and express a Sox10 promoter-driven transgenic reporter that is also expressed in microvillous neurons.^3,28^ Furthermore, work from our group has demonstrated that peridermal cells, which arise from a lineage distinct from the placode and NC,^29^ give rise to multiciliated cells found directly adjacent to OSNs.^30^ Thus, we have much yet to learn about cellular origins as well as new cell types, and perhaps future discoveries will move us past both the single- and dual-origin paradigms of olfactory development.

Regardless of their origins, it is evident that a large number of progenitor cells proliferate, migrate/rearrange, and differentiate over long periods of time to form the olfactory epithelium’s complex, self-renewing neuroepithelial structure. To delineate the fates of a wide range of specialized cells, it is imperative to track and comparatively analyze not just their spatial origins but also their behavior across a large expanse of developmental space and time. Our own unpublished data indicate that olfactory cells exhibit chaotic patterns of motion in which they often do not adhere to stereotypical beginning, intermediate, or final locations and are subject to constant fluctuations in intercellular signaling. Moving forward, it will be important to evaluate how much consideration ought to be given to a common “historical identity” of lineage-traced cells as opposed to the “current identity” of progenitor cells that exhibit similar behaviors. The implementation of system-wide approaches that track and quantitatively analyze multicellular dynamics will help build a clearer picture of how cell lineage connects to terminal identity.

## Data Availability

All data needed to evaluate the conclusions in the paper are present in the paper and/or the Supporting Information.
